# A 2-Deoxyglucose-Resistant Mutant of *Saccharomyces cerevisiae* Shows Enhanced Maltose Fermentative Ability by the Activation of *MAL* Genes

**DOI:** 10.3390/foods7040052

**Published:** 2018-04-01

**Authors:** Yoshitake Orikasa, Dai Mikumo, Takuji Ohwada

**Affiliations:** 1 Department of Life and Food Science, Obihiro University of Agriculture and Veterinary Medicine, Obihiro, Hokkaido 080-8555, Japan; yosori@obihiro.ac.jp; 2 The United Graduate School of Agricultural Science, Iwate University, Morioka, Iwate 020-8550, Japan; mikum@nitten.co.jp; 3 Research Center, Nippon Beet Sugar Manufacturing Co., Inada-cho, Obihiro, Hokkaido 080-0831, Japan

**Keywords:** *Saccharomyces cerevisiae*, catabolite repression, maltose metabolism, *MAL* gene, 2-DOG resistance

## Abstract

*Saccharomyces cerevisiae* MCD4 is a 2-deoxyglucose (2-DOG)-resistant mutant derived from the wild-type strain, AK46, wherein the 2-DOG resistance improves the maltose fermentative ability. In the *MAL* gene cluster, mutations were detected in *MAL11* and *MAL31*, which encode maltose permeases, and in *MAL13* and *MAL33*, which encode transcriptional activators. In maltose medium, the expression of *MAL11* and *MAL31* in MCD4 was 2.1 and 4.2 times significantly higher than that in AK46, respectively. Besides, the expression of *MAL13* and *MAL33* also tended to be higher than that of AK46. Although no mutations were found in *MAL12* and *MAL32* (which encode α-glucosidases), their expression was significantly higher (4.9 and 4.4 times, respectively) than that in AK46*.* Since the expression of major catabolite repression-related genes did not show significant differences between MCD4 and AK46, these results showed that the higher maltose fermentative ability of MCD4 is due to the activation of *MAL* genes encoding two maltose permeases and two α-glucosidases.

## 1. Introduction

Baker’s yeast plays an important role in fermentation for the expansion of bread dough; the production of CO_2_, organic acids and esters by yeast improves dough texture. The identification and characterization of numerous yeast strains [1] have indicated *Saccharomyces cerevisiae* as the main species used in industrial bread production. The primary ingredient of dough is starch, which is hydrolyzed to maltose by the activity of enzymes such as amylase. Thus, a large amount of maltose is released by amylase in the process of dough mixing. Maltose is a disaccharide and is decomposed into glucose by α-glucosidase. Therefore, in the fermentation process for making bread dough, the consumption of maltose as well as glucose is important to produce energy for the metabolism of yeast—particularly for shortening the fermentation time for industrial yeast. The amounts of released maltose are correlated with the expansion of the dough [2], and the maltose promotes the fermentation of sugar-free dough in industrial bread production [3,4].

*S. cerevisiae* harbors a *MAL* locus comprising the *MAL1*, *MAL2*, and *MAL3* genes for maltose metabolism [5,6,7]. *MAL1* encodes maltose permease, which permeabilizes the plasma membrane to allow entry of maltose into cells. The *MAL2* gene product, α-glucosidase (maltase), breaks maltose down into two glucose molecules, whereas *MAL3* encodes a transcription factor that regulates the expression of these two genes. It has been reported that maltose can only be metabolized when all three *MAL* genes are present [5] and that maltose induces their expression, dependent on the absence of glucose and fructose [8]. Moreover, it is considered that yeasts used for industrial applications began to acquire such multiple *MAL* genes during evolution [9,10].

In a previous study, *S. cerevisiae* AK46 was identified as a superior wild yeast strain, based on the characteristics of the bread that it produced [11]. However, AK46 required improvements in fermentative ability prior to application as an industrial yeast. It was reported that 2-deoxyglucose (2-DOG)-resistant mutants increased the leavening ability in bread dough containing maltose, even in the presence of glucose [12]. In addition, 2-DOG-resistant mutants having maltose-deregulated phenotype were reported to improve leavening ability in bread dough [13,14]. Thus, we isolated a 2-DOG-resistant mutant derived from AK46 (termed MCD4) and showed its improved leavening ability in bread dough along with increased maltose fermentation, which was caused by a release from catabolite repression [15]. However, it is not understood how the MCD4 genes associated with the 2-DOG resistance cause the increase of maltose fermentation. In this study, we evaluated the expression levels of genes involved in maltose metabolism (*MAL* genes) and four major catabolite repression-related genes (*MIG1* encoding transcription factor, *SNF1* encoding carbon catabolite derepressing protein kinase, *TUP1* encoding glucose repression regulatory protein, and *CYC8* encoding general transcriptional co-repressor) between MCD4 and AK46 cultured in maltose-containing medium, and the results showed that the improved maltose fermentative ability by MCD4 is attributable to the activation of *MAL* genes encoding two maltose permeases (*MAL11*, *MAL31*) and α-glucosidases (*MAL12*, *MAL32*), and is not likely to be directly involved in the catabolite repression-related genes.

## 2. Materials and Methods

### 2.1. Yeast Strains

*S. cerevisiae* strain AK46 was isolated in a previous study [11]. The 2-DOG mutant strain derived from AK46—MCD4—was constructed as previously described [15].

### 2.2. Investigation of 2-DOG Resistance in MCD4 Haploid Isolates

MCD4 was cultured on Yeast extract Peptone Dextrose (YPD) agar (1.0% yeast extract, 2.0% polypeptone, 2.0% glucose, and 2.0% agar) at 30 °C for 12 h, and then subcultured on sporulation agar (1.0% potassium acetate, 0.1% yeast extract, 0.05% glucose, and 2.0% agar) at room temperature for 1 week. Cell suspension was prepared in 0.1 M phosphate buffer (pH 6.0) containing Zymolyase (Nacalai Tesque, Kyoto, Japan), and after incubation at 30 °C for 1 h, spores were separated using a micromanipulator (Microdissector, Axio Lab, Carl Zeiss, Oberkochen, Germany). The isolates derived from the spores were regarded as MCD4 haploid. The MCD4 haploid isolates were inoculated on maltose minimal agar (2.0% maltose (Wako Pure Chemical Industries, Osaka, Japan), 0.67% Yeast Nitrogen Base without amino acids (Becton, Dickinson and Company, Franklin Lakes, NJ, USA), and 2.0% agar) containing 0.08% 2-DOG and cultured at 30 °C for 10 days. The isolates that could form colonies were determined to be 2-DOG-resistant.

### 2.3. Maltose Fermentative Ability

The fermentative ability in liquid medium was measured as described previously [15]. Briefly, 5 mL cell suspension including yeast cells (200 mg dry weight) was inoculated into 20 mL maltose-medium (8.0% maltose, 0.3% NaH_2_PO_4_∙2H_2_O, 0.2% MgSO_4_∙7H_2_O, 0.08% KCl, 0.002% thiamine, 0.002% pyridoxine, and 0.02% nicotinic acid) in a 100 mL Erlenmeyer flask (HARIO, Tokyo, Japan) and incubated at 30 °C for 3 h aerobically (80 rpm). The weight of the flask was measured immediately before and after the incubation, and the decrease in weight during incubation (i.e., fermentative ability) was calculated.

### 2.4. DNA Sequencing and Identification of Amino Acid Substitutions/Deletions

The total DNA was extracted as described previously [16]. Briefly, yeast cells were incubated in YPD medium at 30 °C for 24 h aerobically and collected by centrifugation at 15,000× *g* for 10 min at 4 °C. The total DNA was extracted from cells using the ZR Fungal/Bacterial DNA kit (ZYMO Research, Irvine, CA, USA) and sequenced as described in a previous study [15]. To identify amino acid substitutions/deletions in proteins associated with maltose metabolism and catabolite repression, the Bioedit multiple sequence alignment editor (Ibis Biosciences, Carlsbad, CA, USA), the BLAST database, and Tablet [17] were used.

### 2.5. Total RNA Extraction

Yeast colonies formed on YPD agar were inoculated into YPD liquid medium in a 3-mL test tube and cultured aerobically on a reciprocal shaker at 150 rpm and 30 °C for 24 h. A portion of the culture (0.6 mL) was transferred to 60 mL Yeast extract Peptone Sucrose (YPS) medium (2.0% Bacto Yeast Extract, 4.0% Bacto Peptone, 2.0% sucrose, 3.0% NaCl, 0.2% KH_2_PO_4_, and 0.1% MgSO_4_·7H_2_O) in a 300-mL baffled conical flask with a silicone stopper and cultured aerobically on a rotating shaker at 150 rpm and 30 °C for 24 h. Cells were harvested by centrifugation and washed twice with distilled water. Then, the yeast cells were incubated for 1 h in sucrose-medium (10.0% sucrose as the carbon source, 0.3% NaH_2_PO_4_∙2H_2_O, 0.2% MgSO_4_∙7H_2_O, 0.08% KCl, 0.002% thiamine, 0.002% pyridoxine, and 0.02% nicotinic acid) or maltose-medium. Total RNA was extracted from yeast samples using the RNeasy Mini kit (Qiagen, Valencia, CA, USA).

### 2.6. Quantitative Reverse Transcription-Polymerase Chain Reaction (RT-PCR)

The total RNAs were extracted as described above and the primers were designed using Primer3Plus software [18]. Primer oligonucleotides used in this experiment are shown in Table 1. Total RNAs (50 ng) were used as a template and the quantitative RT-PCR analyses were conducted on an Applied Biosystems 7300 Real-Time PCR System (Thermo Fisher Scientific K.K., Yokohama, Japan) in combination with the High Capacity cDNA Reverse Transcription kit and Power SYBR Green PCR Master Mix (Applied Biosystems, Foster City, CA, USA). Quantification was conducted according to the real-time PCR experiment guide provided by Hohjoh [19], and the results were normalized using expression levels of the glyceraldehyde-3-phosphate dehydrogenase (GAPDH) gene (*TDH1*) [20]. The standard curve of Δ*C*_t_ and mRNA of *TDH1* were used for normalizing the expression levels of MCD4 and AK46 genes.

## 3. Results and Discussion

### 3.1. 2-DOG Resistance Improves Maltose Fermentative Ability

**Figure 1:**
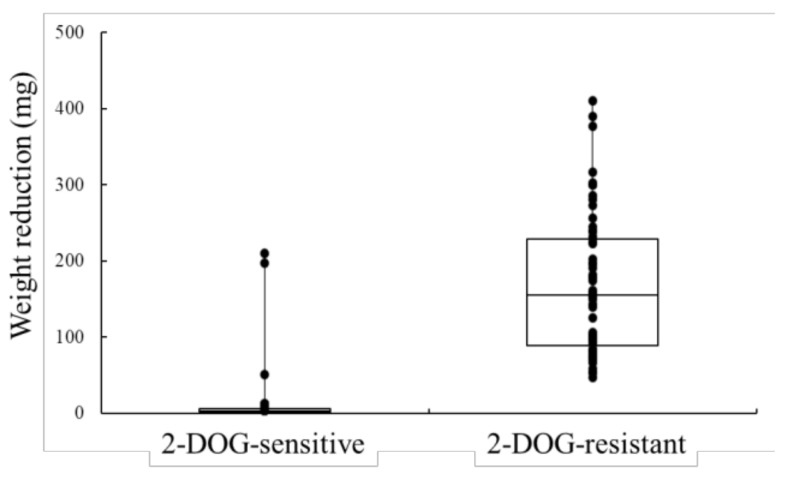
Distribution of the weight reduction (maltose fermentative ability) of 2-deoxyglucose (2-DOG)-sensitive and -resistant haploids isolated from MCD4 as box plots. Vertical axis shows the decrease in weight during incubation for 3 h, as described in the Materials and Methods Section 2. Closed circles indicate weight reduction of each isolate.

*Saccharomyces cerevisiae* MCD4 with 2-DOG-resistance showed a higher ability of maltose fermentation compared with the parent strain AK46 as well as a standard baker’s yeast HP216, which was used as a comparative control [15]. Therefore, to investigate the relationship between 2-DOG resistance and maltose fermentative ability, a total of 120 haploid isolates of MCD4 were obtained, and their growth abilities in the 2-DOG-containing medium and fermentative abilities (reduction in total weight by the CO_2_ production) in maltose-medium were evaluated (Figure 1). As a result, 57 out of 58 isolates with 2-DOG resistance showed more than 50 mg of weight reduction over the 3-h fermentation period, and particularly, 18 isolates showed more than 200 mg of weight reduction (median and mean value: 155 and 167 mg, respectively). In contrast, 60 out of 62 isolates with 2-DOG sensitivity showed a weight reduction of less than 50 mg (median and mean value: 3.55 and 10.98 mg, respectively) (Figure 1). These results demonstrate that the maltose fermentative ability is correlated with the 2-DOG resistance.

### 3.2. 2-DOG-Resistant Mutant MCD4 Activates MAL Genes

**Figure 2:**
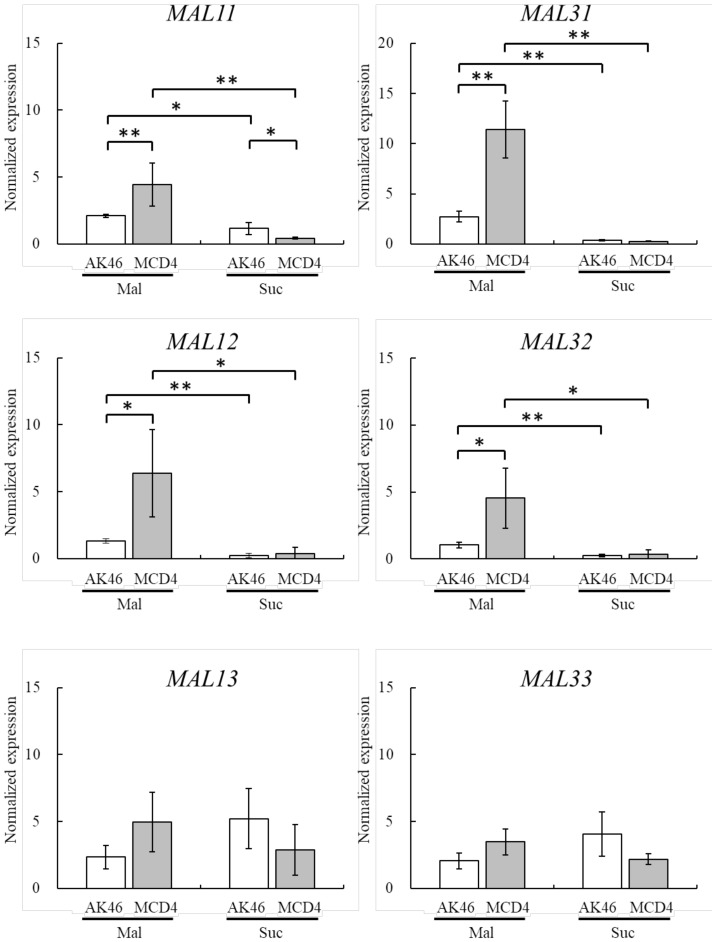
Relative expression of maltose metabolism-related genes (*MAL11*, *MAL12*, *MAL13*, *MAL31, MAL32*, and *MAL33*) in *S. cerevisiae* AK46 and MCD4 incubated in maltose- or sucrose-medium. The values are shown as the means ± S.D. (standard deviation) of three replicates. Mal: maltose, Suc: sucrose. * *p* < 0.05; ** *p* < 0.01 (Student’s *t*-test).

Next, we determined the expression of six genes related to maltose metabolism (*MAL11* and *MAL31*, which encode maltose permeases; *MAL12* and *MAL32*, which encode α-glucosidases; and *MAL13* and *MAL33*, which encode transcriptional activators) by quantitative RT-PCR in maltose-medium and sucrose-medium as a reference (Figure 2). When MCD4 and AK46 cells were cultured in maltose-medium, the expression of all the genes except *MAL13* and *MAL33* was significantly higher than that in sucrose-medium, indicating that almost all *MAL* genes were induced in the presence of maltose (Figure 2). In maltose-medium, the expression of *MAL11* and *MAL31* in MCD4 was significantly higher (2.1- and 4.2-fold) than that in AK46, respectively (Figure 2). Notably, a previous study showed that the amino acid sequence of both Mal11 and Mal31 was changed at some positions, although disruption of the gene products appeared not to occur in MCD4 (Table 2) [15]. Therefore, it might be possible that the increased expression resulted from substitutions and/or deletions of amino acids in the transcription domain or structure of these gene products. It has been reported that the maltose metabolism was increased when maltose could be readily taken up by cells through increased expression of a gene encoding maltose permease [21], suggesting that maltose permease is important for the metabolism of maltose in yeast. Moreover, in the yeast *Kluyveromyces*, the loss of catabolite repression has been attributed to the reduction of glucose uptake following the deletion of the gene encoding hexose permease [22].

The expression of genes encoding α-glucosidases—*MAL12* and *MAL32* in MCD4—was also significantly higher than that in AK46, reaching approximately 4.9- and 4.4-fold of that in AK46 in maltose-medium, respectively (Figure 2). Previously, we reported that α-glucosidase activity was 1.6-fold higher in MCD4 than in AK46 [15], indicating that the increased transcription of *MAL12* and *MAL32* resulted in an increased α-glucosidase level. It has been reported that the expression level of gene encoding α-glucosidase correlated with its enzyme activity [21]. α-glucosidase is also an essential enzyme for maltose metabolism in yeast; for example, maltose fermentation in dough is increased by overexpression of the genes encoding permease and α-glucosidase [21]. The expression of *MAL13* and *MAL33* in AK46 tended to be lower in maltose-medium than in sucrose-medium. However, the levels of these genes in MCD4 had a tendency to be higher in maltose-medium than in sucrose-medium. Besides, the expression of *MAL13* and *MAL33* in MCD4 tended to be higher compared with that in AK46 in maltose-medium. These results suggest that *MAL13* and/or *MAL33* in MCD4 may act as a transcriptional activator for *MAL* genes such as *MAL11*, *MAL31*, *MAL12,* and *MAL32* in maltose-medium (Figure 2).

Transcription factors such as Mal13 and Mal33 are well-studied in galactose metabolism. In the conventional Gal4 model, the transcription factor Gal4 binds to the upstream activation sequence (UAS) for the regulation of galactose metabolism [24]. Similarly, Mal13 has been reported to activate the transcription of *MAL11* and *MAL12* through its association with UAS [25]. Results of *MAL13* nucleotide sequencing revealed a zinc finger (ZnF) motif, which serves a wide variety of biological functions such as DNA binding [26] in the Gal4-homologous domain. Although the C-terminal zinc finger showed no base substitutions, the N-terminal zinc finger exhibited three amino acid substitutions (R336W, R337H, and I341V) (Table 2). These results suggest that mutations in the ZnF domain of *MAL13* may cause an enhancement of maltose-inducible expression of the *MAL* structural genes. In comparison, mutations in the C-terminal region of the transcription activator MAL63—containing a DNA-binding region, functional core region, and inhibitory region of activation function [27]—lead to constitutive *MAL*-activation [28].

### 3.3. Involvement of Catabolite Repression-Related Gene in the Improved Maltose Fermentation of MCD4

**Figure 3:**
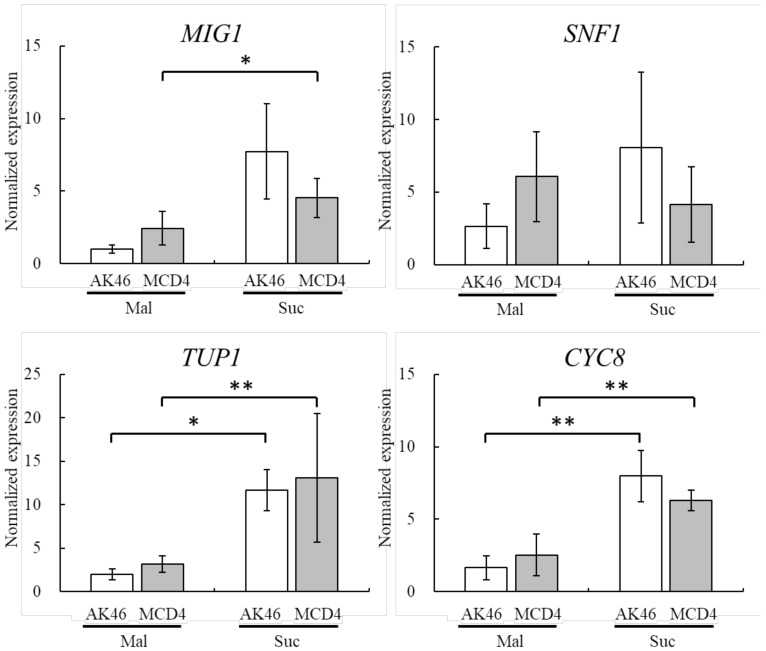
Relative expression of catabolite repression-related genes (*MIG1*, *SNF1*, *TUP1*, and *CYC8*) of *S. cerevisiae* AK46 and MCD4 incubated in maltose- or sucrose-medium for 1 h. The values are shown as the means ± S.D. of three replicates. * *p* < 0.05; ** *p* < 0.01 (Student’s *t*-test).

Next, in order to elucidate if MCD4 genes involved in catabolite repression relate to the increased maltose fermentative ability, we quantified the expression of four major catabolite repression-related genes (*MIG1*, which encodes a transcription factor; *SNF1*, which encodes a carbon catabolite derepressing protein kinase; *TUP1*, which encodes a glucose repression regulatory protein; and *CYC8*, which encodes a general transcriptional co-repressor) in maltose-medium and sucrose-medium (control) (Figure 3). It was reported that the Cyc8 (Ssn6)-Tup1 complex—which is composed of one Cyc8 and four Tup1 subunits—is required in regulatory systems such as glucose repression in yeast cells, although the expression of *CYC8* alone may not affect this complex formation directly [29]. However, in MCD4 as well as AK46, the expression levels of both *TUP1* and *CYC8* were significantly lower in maltose-medium than in sucrose-medium. In addition, Mig1 is known to have a Cys2His2 zinc finger motif and act as a glucose repressor through the formation of a Mig1-Cyc8-Tup1 complex, although the increase in the expression of *MIG1* alone may not play an important role in glucose repression [30], and Snf1 is known to play an important role in dough fermentation as a Ser/Thr protein kinase [31,32,33]. However, the expression of *MIG1* and *SNF1* (except SNF1 in MCD4) also tended to be lower in maltose-medium than in sucrose-medium for both MCD4 and AK46 (Figure 3). Besides, there were no significant differences between AK46 and MCD4 for the expression of these four genes (Figure 3). The results seem to suggest that these major catabolite repression-related genes are not inducible in maltose-medium and are not responsible for the enhancement of the maltose fermentative ability due to the activation of *MAL* genes.

In addition to these four major genes, substitutions/deletions of amino acids translated from the other four catabolite repression-related genes (*GRR1*, which encodes ubiquitin-ligase; *REG1*, which encodes protein phosphatase; and *HXT2/4*, which encodes a high-affinity glucose transporter) of MCD4 were also studied. Notably, no amino acid substitutions/deletions except for Cyc8 and Grr1 were detected, although *REG1* and *HXT4* harbored some silent mutations in the nucleotide sequence (Table 2). A three-residue deletion of consecutive asparagines (amino acids 44 to 46) was found in Grr1 (Table 2). It was reported that Grr1 contains a leucine-rich motif to mediate protein-protein interactions; however, this three-residue deletion did not appear to have an effect on protein interactions [34]. Reg1 is known as an enzyme that dephosphorylates Mig1 for the formation of the Mig1-Cyc8-Tup1 complex. In addition, Lin et al. reported that the *REG1* mutation showed an improvement of maltose metabolism in yeast [35]. However, no amino acid substitutions and/or deletions in Reg1 of MCD4 were detected (Table 2). Hxt2 and Hxt4 are known to form a high-affinity glucose transporter that is expressed only at low glucose concentrations, as *HXT2* and *HXT4* expression is suppressed by Mig1 via catabolite repression at high glucose concentrations [36]. However, our results showed no amino acid substitutions in either protein in MCD4 (Table 2).

Results obtained here show that the improved maltose fermentative ability of MCD4 is attributable to the activation of both *MAL11* and *MAL31*, which encode maltose permeases, and *MAL12* and *MAL32*, which encode α-glucosidases, and some mutations in *MAL* activators (i.e., *MAL13* or/and *MAL33*) may cause the enhancement of maltose-inducible expression of these *MAL* structural genes (Figure S1). However, it still remains unclear how catabolite repression-related genes are involved in the improved maltose fermentative ability. Further investigations are needed to elucidate how the 2-DOG resistance results in the activation of these *MAL* structural genes, followed by the production of Mal proteins in MCD4.

## 4. Conclusions

The expression of *MAL11* and *MAL31* in 2-DOG-resistant mutant (MCD4) was significantly higher than that in wild-type strain (AK46). Additionally, *MAL12* and *MAL32* expression was significantly higher than that in AK46. These results showed that the higher maltose fermentative ability of MCD4 is due to the activation of *MAL* genes encoding two maltose permeases and two α-glucosidases.

## Figures and Tables

**Table 1:** Oligonucleotide primer sequences used in this study.

Gene	Orientation	Sequence (5′→3′)
Maltose metabolism		
*MAL11*	Forward	GTCTTGGGTTAGCGGGTACA
	Reverse	CAACTCCGCTGATGGAATTT
*MAL12*	Forward	AAGGTATCACTTCCAAGTTGC
	Reverse	AGTCCTCATTGGTACCTATG
*MAL13*	Forward	GCAACCGTCGAGAAAAAGAG
	Reverse	ATAGAGCCGCAAGCACTGAT
*MAL31*	Forward	GTAGCCATGGGGTTGTTTC
	Reverse	CAGATCCACTGCAAAGCAAA
*MAL32*	Forward	ACATACGGTACCAACGAGGA
	Reverse	GTTTGCGAGTCGTCAAGTTG
*MAL33*	Forward	ATGAAGTTGGAGGCTTGGAA
	Reverse	ATCATTTAGGCGCAGTGGTC
Catabolite repression		
*MIG1*	Forward	GGTTGTGGGCTCTCCAATAA
	Reverse	CCATCGTTTTGGGAGAAGAA
*SNF1*	Forward	TACCACTACGGGCCAAAAAG
	Reverse	CCCGGCGTACTCTATAACCA
*TUP1*	Forward	AAGGACGCGTACGAAGAAGA
	Reverse	GCAACTGGAACAGATGCAGA
*CYC8*	Forward	GCCAAAGTTTTGGAATTGGA
	Reverse	CATGCTCGTAGGCTTCCTTC
Normalization		
*TDH1*	Forward	CTCTACCGGTGCTGCTAAGC
	Reverse	AACGGCATCTTCGGTGTAAC

**Table 2:** Amino acid substitutions/deletions in maltose metabolism and catabolite repression-related proteins of MCD4.

Protein	Description	Amino Acid substitutions *	Reference
Maltose metabolism			
Mal11	sugar transporter	H591L/D592I/S593R/I594X **	[15]
Mal12	α-glucosidase	none	[15]
Mal13	activator	T299I/T318A/S320X/N327Y/T330I,V/S333A/R336W/R337H /I341V/N361R/G362A/Q363H/I364V/R370S/E381D,K,N /D385E,G/V391I/V393A,I,M,T/T395A/L396I/I398V/T400N	[15]
Mal31	sugar transporter	H49R/A122S/S146P/Q166H/M175L/Q261T/A265P /E268N/E339K/T349S/V354L/G357S/I358V/C374I,S /S375T/A378T/S379P,Q,X/S394G/V508A,I,T/T509R,S /K526L/F534L/L536F/A540V/V544I	[15]
Mal32	α-glucosidase	none	[15]
Mal33	activator	S240A/V243I/H244D,K,Q/Q257L/F260V/D269E/F272L /M274V/F286Y/E292V/K305R/K308N/A313T/L315H /E316D/I327F/F329C/S330F,L/H332P/A336T/F343L /Q344H/N346K/K365R/D369E,G/I371M,T/S390A/V393I /K403Q/Y404H/H406K,N,Q	[15]
Catabolite repression			
Mig1	transcription factor	none	[15]
Snf1	carbon catabolite derepressing protein kinase	none	[15]
Tup1	glucose repression regulatory protein	none	[15]
Cyc8	general transcriptional co-repressor	Q26R,Q/Q28R,Q	This study
Grr1	ubiquitin-ligase	N44N,-/N45N,-/N46N,-	This study
Reg1	protein phosphatase	none	This study
Hxt2	high-affinity glucose transporter	none	This study
Hxt4	high-affinity glucose transporter	none	This study

* Amino acid substitutions are shown in the format (original amino acid) (position of amino acid) (substituted amino acid(s) and/or a stop codon, X and/or unspecified,-) according to the description of Dunnen and Antonarakis [23]. Substituted amino acid(s) include original amino acids except for Mal11. ** Amino acid deletion downstream to the 593rd by frame shift at the 1772nd nucleotide

## Supplementary Materials

Supplementary material is available online at http://www.mdpi.com/2304-8158/7/4/52/s1, Figure S1: The functional roles of 6 *MAL* genes and Mal proteins in *S. cerevisiae* AK46 and MCD4 on maltose metabolism.
