# Supplementary material for: A 2-Deoxyglucose-Resistant Mutant of Saccharomyces cerevisiae Shows Enhanced Maltose Fermentative Ability by the Activation of MAL Genes
Source: Foods. 2018 Apr 1;7(4):52. doi: 10.3390/foods7040052 (PMC5920417; doi:10.3390/foods7040052)
Supplement: Supplementary file 1 [file foods-07-00052-s001.pdf]

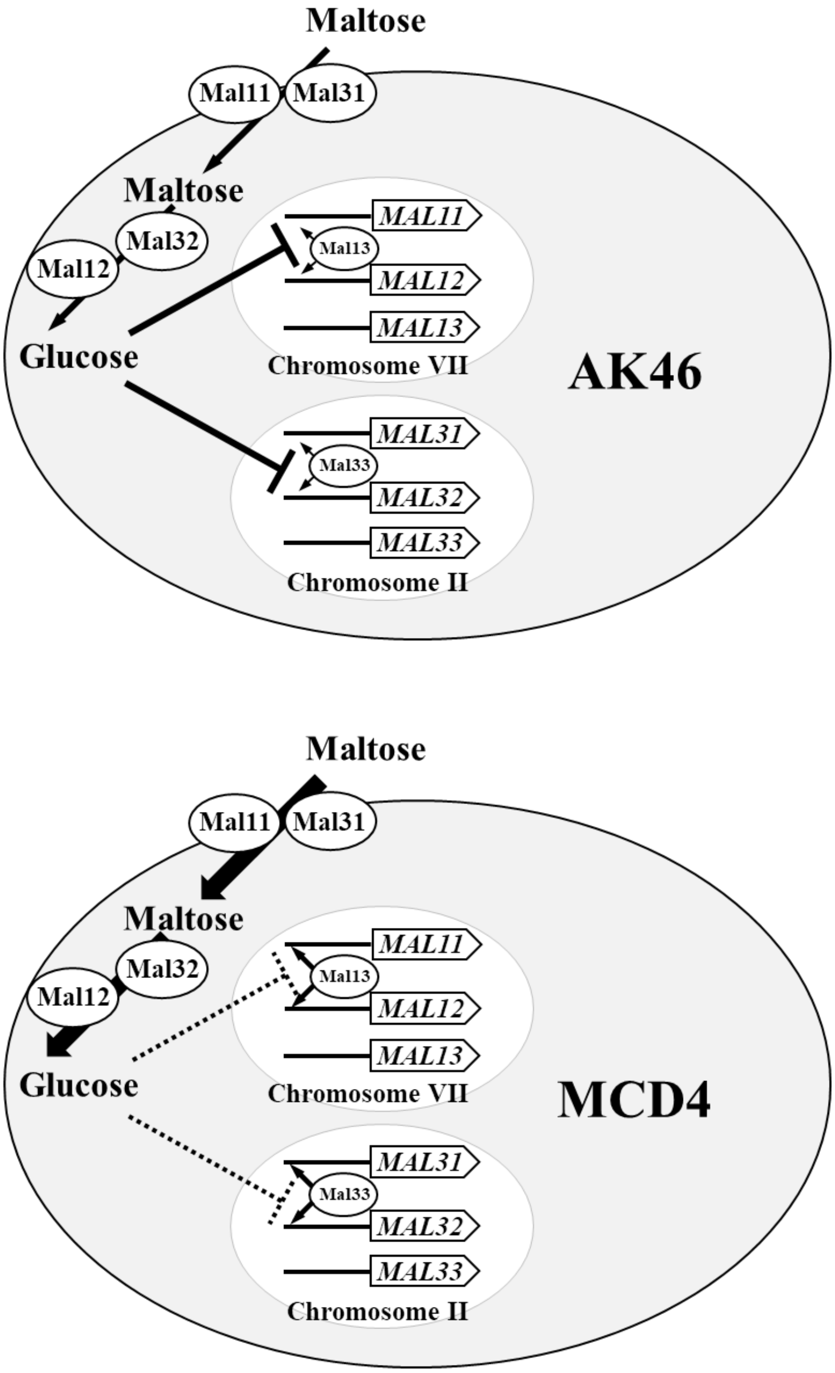

Figure S1. The functional roles of 6 *MAL* genes and Mal proteins in *S. cerevisiae* AK46 and MCD4 on maltose metabolism. Mal11 and Mal13, maltose permease; Mal12 and Mal32, α-glucosidase; Mal13 and Mal33, *MAL* gene activator. Thick arrows show the enhancement of gene transcription or function of Mal proteins. Broken lines indicate the release from catabolite repression.
